# Letter to the Editor: CRISPR-based gene editing for cardiac protection in Barth syndrome

**DOI:** 10.1097/MS9.0000000000004188

**Published:** 2025-10-27

**Authors:** Zain Ul Abedin, Anzah Imtiaz Waggan, Esha Khan, Muhammad Umer Suleman, Syeda Nafisa Tabassum

**Affiliations:** a King Edward Medical University, Lahore; b Jinnah Sindh Medical University, Karachi, Pakistan; c Nishtar Medical University, Multan; d Ayub Medical College, Abbottabad, Pakistan; e Department of Microbiology, Bangladesh Rural Advancement Committee: BRAC, Dhaka, Bangladesh

**Keywords:** adeno-associated virus vectors, barth syndrome, cardiolipins, cardiomyopathy, dilated, crispr-cas systems, gene editing, gene therapy, induced pluripotent stem cells, mitochondrial diseases, myopathy, neutropenia

## Abstract

Barth syndrome is a rare X-linked mitochondrial disorder caused by mutations in the Tafazzin (TAZ) gene. These mutations make it hard for cardiolipin to remodel and mitochondria to work properly. This condition is characterized by growth retardation, neutropenia, skeletal myopathy, and dilated cardiomyopathy, frequently leading to significant morbidity and mortality, with numerous patients necessitating heart transplants. There are no treatments available at this time to fix the genetic problem. Recent progress in gene editing, especially CRISPR-based methods, holds great promise for fixing TAZ mutations. Research utilizing patient-derived cardiomyocytes has demonstrated that the rectification of TAZ mutations reinstates mitochondrial efficiency and enhances cellular functionality. Animal models, including TAZ-knockout mice, have exhibited substantial enhancements in cardiac function, survival rates, and diminished fibrosis subsequent to gene replacement therapy.

To the Editor,

Barth syndrome is an extremely rare X-linked mitochondrial disease. It is caused by mutations in the Tafazzin (TAZ) gene. Mutations in TAZ impair the remodeling of cardiolipin, an essential phospholipid that maintains the integrity and functionality of the mitochondrial membrane. Growth retardation, neutropenia, skeletal myopathy, and dilated cardiomyopathy are among the clinical manifestations, which often appear in early childhood. Despite improvements in supportive care and the treatment of heart failure, morbidity and mortality remain high. Some patients need a heart transplant, while the majority suffer from progressive cardiac decline.^[1]^ Unfortunately, there is no treatment that addresses the underlying genetic abnormality at this time. It emphasizes the need for novel approaches that can change how this life-limiting illness progresses.

In recent years, modifying genes has emerged as a novel method for directly correcting the genetic changes in Barth syndrome. In 2025, studies using patient-derived cardiomyocytes demonstrated that accurate correction of TAZ mutations restored mitochondrial efficiency and cardiolipin remodeling.^[2]^ Importantly, these treatments normalized biochemical abnormalities and improved contractile function and cellular energy metabolism. Additionally covered in the article are stem cell models with TAZ deficiency. It exhibits abnormal mitochondria and altered cardiomyocyte stress responses, phenotypes that could be treated by molecular therapies like genome editing.^[2]^

Experiments on animals have also been reported using this technique. Wang *et al*^[3]^ demonstrated that adeno-associated virus (AAV)-mediated human TAZ gene replacement treatment in TAZ-knockout mice prevented neonatal mortality once sufficient cardiomyocyte transduction was achieved. It improved cardiac function and reversed fibrosis and established cardiomyopathy. The treated animals showed reduced signs of heart failure and better survival. Such outcomes demonstrate that genomic repair can yield meaningful physiological benefits, offering a pathway toward durable cardiac protection.

One of the main challenges in advancing this work is the rarity of the disease. Barth syndrome affects fewer than 1 in 300 000 live births, making large clinical trials very difficult.^[4]^ However, its well-defined monogenic origin makes it an ideal candidate for translational gene editing approaches. Beyond Barth syndrome, lessons from these efforts may serve as a model for other mitochondrial and inherited cardiomyopathies, thereby broadening the reach of therapeutic innovation.

Looking forward, the key step is to translate these findings into carefully designed clinical trials. Early-phase human studies must establish the feasibility, safety, and durability of TAZ correction. Given the limited patient population, international registries, natural history studies, and adaptive clinical trial designs will be critical to maximize data collection and accelerate development. Ethical oversight and long-term monitoring will also be essential to mitigate risks associated with first-in-human genome editing.^[5]^

In summary, CRISPR-based editing of TAZ mutations represents a transformative opportunity to move Barth syndrome care beyond palliation toward disease modification. With encouraging results already seen in both cell and animal studies, the time has come to consider clinical trials. If successful, this approach could not only improve outcomes for patients with Barth syndrome but also open new doors for treating other rare inherited cardiomyopathies. Our work is in line with the TITAN Guidelines on the need for transparency in AI use in healthcare^[6]^.

## Data Availability

Data sharing does not apply to this article as no datasets were generated during the current study; all data were sourced from published literature.
